# Independent set-based multivariate graph polynomials for fractal-type silicate triangle structures

**DOI:** 10.3389/fchem.2026.1864298

**Published:** 2026-07-06

**Authors:** K. S. Nithiya, D. Easwaramoorthy

**Affiliations:** Department of Mathematics, School of Advanced Sciences, Vellore Institute of Technology, Vellore, Tamil Nadu, India

**Keywords:** fractal analysis, independence polynomial, independent set, multivariate independence polynomial, self-similar graph, silicate triangle graph

## Abstract

Silicate structures are the mineral materials widely studied for their extraordinary structural complexity and versatility. They naturally exhibit self-similarity, making them well-suited for analysis through chemical graphs, graph polynomials, and fractal theory. Fractals, characterized by their self-replicating patterns and intricate structures, have found extensive applications across various domains, particularly in the study of graphical structures. Independence polynomials serve as mathematical representations of molecular and chemical graph structures and have proven to be a powerful tool for analyzing the intricate structural adjacency relationships in graph molecular structures. This article explores the fractal-type silicate triangle graph, characterized by recursive and iterative patterns, as a distinguished class of self-similar graphs. An innovative computational approach is presented for evaluating their independence polynomials and multivariate independence polynomials at specific iterations n=1,2 and 3. This proposed algorithm systematically identifies independent sets and computes the corresponding single-variable, multivariable polynomials and its inverse by employing SAGE mathematical software. A graphical analysis is performed for specific iterations, providing deeper insights into the combinatorial and structural characteristics of these representative graphs.

## 1 Introduction

Silica minerals are the most abundant and geologically significant minerals found in the earth’s crust, both in high- and low-temperature hydrous environments. They play an important role in understanding mineral formation, the plate tectonics, and Earth’s evolutionary history (Monger and Kelly, 2002). One of the most familiar crystalline forms of silica is quartz (Gotze, 2018). Silica minerals are mainly composed of silicon dioxide $\left(SiO_{2}\right)$ and may also contain sodium, potassium, magnesium, iron, calcium, and aluminum, making them structurally more complex and expanding their range of functions (Easton, 1972; Samantray et al., 2022). Silica plays an essential role in industrial applications. Almost 95 percent of silica sand is used commercially in the construction industry to make concrete, mortar, and bricks. High-purity silica is used to make durable transparent glass, while crystalline quartz is used in electronics because of its piezoelectric properties (Shaukat et al., 2023; Zhang et al., 2018). Silica is also used in the manufacture of porcelain, heat-resistant materials, water filtration systems, and sandblasting works (Nayl et al., 2022).

The basic structure of silicate minerals is the silicon-oxygen tetrahedron, $SiO_{4}$ , which is composed of four oxygen atoms surrounding a single silicon atom. The tetrahedra may be isolated or linked together in chains, sheets, and three-dimensional frameworks to produce complex silicates such as pyroxenes, micas, and feldspars (Deer et al., 2013; Masai et al., 2023). The tetrahedra form various silicate networks, including chain, cyclic, hexagonal, rhombic, and trapezoidal structures, which are used to classify the silicate minerals (Hawthorne et al., 2019). Detailed studies on silicate structures are discussed in Arockiaraj et al. (2018), Irfan et al. (2021), Das et al. (2023), and Khan et al. (2023). Synthetic analogs of silicate structures inspire the design of new type of materials, such as zeolites, which are used in catalysis and molecular sieving (Peter et al., 2025; Jacob and Clement, 2024). Silicate minerals, particularly zeolites, are also utilized in environmental applications, including water filtration and air pollution management, helping to reduce environmental contaminants and improve water quality (Jiang et al., 2018).

Mathematical chemistry is a discipline that employs mathematical methods to solve problems in chemistry. Chemical graph theory is one of its central branches, investigating the structural and behavioral characteristics of molecular structures. To process research in this area, a conversion is needed to generate a graph with vertices representing the atoms in the chemical complex structure and edges reflecting the bonds between them. A graph G is made up of vertices V(G) or atom groupings that are connected by edges E(G) or bonds. Chem-informatics combines information science with mathematics and chemistry. These combinations are used to explore certain physicochemical features of molecules and derive mathematical results (Zhang et al., 2025). Graph theory employs various polynomials, including independence, characteristic, chromatic, matching, Hermite, Tutte, and M-polynomials as mathematical tools to analyze and quantify the structural behavior and other properties of graph networks. Every polynomial represents a particular geometric configuration and provides a systematic method for examining basic structural properties. In applications such as modeling parabolic curves and finding extrema of functions, quadratic polynomials play a vital role, as discussed by Mufti et al. (2023). Independent sets and independence polynomials have also been used to construct biclique polynomials that describe the relationships between different graph structures (Lumpayao et al., 2024).

The independence polynomial (IP) is well studied due to its wide applications and theoretical importance, especially in chemical graph theory, where it is used to describe the structural and stability properties of molecular graphs. This study is well connected with the fields of combinatorics, computational complexity, phase transition theory, and statistical physics (Valiant, 1979). Furthermore, graph polynomial analysis can be used to understand the structural and dynamic behaviors of complex networks, connecting abstract mathematical concepts to real-world networks (Galvin et al., 2024; Bencs et al., 2023). Independent sets have important applications in the study of interactions and steric stability in molecular structures, and the independence polynomial is an important measure of the number of independent sets of different sizes, providing insight into stable molecular configurations (Leake and Ryder, 2019; Harvey et al., 2018). It also provides a quantitative measure of molecular complexity and is useful in isomer analysis as an invariant to differentiate structurally different molecules that have the same molecular formula. In addition, IP can be used to count different parts of a molecule or sub-structures in large chemical graphs, such as polymers, thereby aiding the study of how microscopic structure relates to macroscopic properties.

Multivariate Independence Polynomial (MIP) plays a vital role in mathematical chemistry as they provide a graph-theoretical way of describing the structural complexity of molecules. MIPs function as a useful method for studying the relationships between independent sets of vertices of different sizes. The MIP allows a more detailed analysis of self-similarity by incorporating several variables as it captures the relationships among variables associated with independent sets. This multivariate approach reveals complex structural properties that may remain hidden when using a single-variable polynomial (Nithiya and Easwaramoorthy, 2024). These polynomials serve as useful tools for understanding and predicting complex molecular processes and their applications in environmental chemistry, materials engineering, and drug development (Balasubramanian 2019, 2022).

The evolution of fractal geometry is quite fascinating in pure and applied mathematics, especially in the field of self-similar structures and their network. This revolutionary concept was first introduced by Benoit Mandelbrot in 1975 through his nature-inspired geometric approach and established a platform for independent research (Mandelbrot, 1983; Barnsley, 2014). The development of fractal theory was greatly advanced by mathematicians such as Besicovitch, Cantor, Julia, Hausdorff, Menger, Henri Poincare, Sierpinski, Von Koch, Weierstrass, and many others (Addison, 1997; Banerjee et al., 2021). Fractals are geometric objects that are exactly self-similar, with a Hausdorff dimension greater than their topological dimension (Falconer, 2004). This self-similarity is expressed in the repeating patterns that are inherent in fractal structures.

Iteration is a fundamental key to establish self-similarity, especially in the context of defining recursively specified sets. In mathematics, the Triadic Cantor set, the Von Koch curve, the Sierpinski gasket, and the Sierpinski triangle are well-known fractal structures. The German mathematician George Cantor created the Cantor set by successively removing parts of the interval of unit length [0,1]. The process of elimination continues indefinitely and gives rise to a set whose dimension is fractional, making it a fractal (Edger, 2013; Schroeder, 2009). In 1904, Swedish mathematician Helge von Koch introduced the Koch curve, which represents a continuous curve that is nowhere differentiable. The Sierpinski gasket was first introduced in early 1915 by Waclaw Sierpinski. The Sierpinski gasket is primarily recognized for its self-similar fractal structure and is characterized by scaling invariance. It is commonly referred to as the Sierpinski gasket or Sierpinski triangle and stands as an iconic example in fractal geometry (Klavzar and Moharm, 2005; Chen et al., 2019). These fractal structures offer more aesthetic appeal, provide valuable insights into the self-similar properties of networks, and have contributed to advancements in graph theory, complex networks, and the development of recursive algorithms (Hinz et al., 2017). This type of mathematical construction demonstrates the profound implication of iterative processes in generating complex geometric structures with fascinating mathematical properties.

In this article, we examine a triangular framework, which is modeled by a Sierpinski Triangle-type fractal structure. This Silicate Triangle graph structure of dimension n is represented by the notation $SiT_{n}$ . A tetrahedron having a silicon atom at the center and oxygen atoms at the corners in a 2D plane that assembles a Silicate Triangle framework is taken. Over the years, researchers have been investigating two-dimensional silicate frameworks across a range of fields to broaden their potential applications (Arockiaraj et al., 2024). Figure 2 illustrates the fundamental structural pattern of the Silicate Triangle graph framework in two dimensions. This work investigates the development of an algorithmic approach for computing independent sets of the representative Silicate Triangle graph ( SiT in short) and for formulating its independence polynomials and multivariate independence polynomials for iterations n=1,2 , and 3. A graphical analysis is undertaken, thereby providing deeper insights and revealing further information about the combinatorial and structural properties of these self-similar chemical graphs.

The following framework describes the structure of this research article. The prerequisites are provided in Section 2. The construction of the Silicate Triangle graph $SiT_{n}$ , its iterations, the IP, and the MIP for iterations of the Silicate Triangle graph are all subjected to a thorough analysis in Section 3. Section 4 presents the graphical depiction of the analysis of IPs and MIPs for the iterations of $SiT_{n}$ for n=1,2 , and 3. The conclusion is briefed in Section 5.

## 2 Graph polynomials

The basic definitions and theorems necessary for this work are recalled in this section. The set of all integers is denoted by Z . The sets of all non-negative integers and positive integers are denoted by the symbols $Z_{+}$ and N , respectively. The cardinality of a finite set S is denoted by |S| . For a given tuple $\underline{q}=\left(q_{1},\ldots ,q_{n}\right)$ of non-negative integers, let $|\underline{q}|=\sum_{i=1}^{n}q_{i}$ . In this section, we review the basic facts about finite simple graphs and the associated IPs and MIPs.

Definition 1 Let G be a finite simple graph, and it is defined as a tuple G=(V,E) , where V denotes a finite set of vertices and E⊆V×V is a set of edges such that (a,a)∉E for all a∈V . This means that the graph contains no multiple edges and no loops. The cardinality of vertices in G is denoted by n (Zhang and Chartrand, 2006).

We refer to V (respectively, E ) as the set of all vertices (respectively, the set of all edges) of G . If e=(a,b)∈E , we say that e is an edge of G connecting the vertices a and b .

Definition 2 Let I be a subset, where I⊆V is referred to as an independent set in a graph G if no pair of vertices a,b∈I are connected by an edge, meaning that (a,b)∉E . We denote the collection of all independent sets of G by I(G) . The independence polynomial of G , denoted by $IP_{G}\left(x\right)$ , is defined as follows (Sreeja et al., 2021; Nithiya and Easwaramoorthy, 2024):

$$IP_{G}\left(x\right)≔\overset{n}{\underset{k=0}{\sum }}c_{k}x^{k},$$

where $c_{k}≔|\left\{I\in I\left(G\right):|I|=k\right\}|$ . It is important to note that by definition, $c_{0}=1$ and $c_{1}=|V|$ .

Definition 3 Let us consider a field F , and let $F\left[x_{1},\ldots ,x_{n}\right]$ represent the polynomial ring with n algebraically independent variables $x_{1},\ldots ,x_{n}$ . The MIP of a graph G , denoted by $MIP_{G}\left(x_{1},\ldots ,x_{n}\right)$ , is a polynomial having the variables $\left(x_{1},x_{2},\ldots ,x_{n}\right)$ and is provided as follows (Cartier and Foata, 2006; Nithiya and Easwaramoorthy, 2024):

$$MIP_{G}\left(\underline{x}\right)=MIP_{G}\left(x_{1},\ldots ,x_{n}\right)=\underset{I\in I\left(G\right)}{\sum }\underset{i\in I}{\prod }x_{i},$$

where the summation runs over all independent sets I of G .

From the multivariable version, we simply fix $x_{i}=x$ for every variable i in the vertex set V of the graph to derive the single-variable independence polynomial $IP_{G}\left(x\right)$ .

**FIGURE 1:**
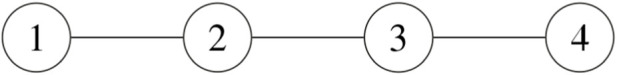
Path $P_{4}$ .

Example 1 For the given path graph $P_{4}$ , as shown in Figure 1, the IP and the MIP are formulated as follows.

$$\begin{array}{ll} IP_{P_{4}}\left(x\right) & =\;1+4x+2x^{2}\\ MIP_{P_{4}}\left(\underline{x}\right) & =\;1+x_{1}+x_{2}+x_{3}+x_{4}+x_{1}x_{3}+x_{2}x_{4}. \end{array}$$

## 3 Construction of Silicate Triangle graph and its iterations

This section recalls the construction of the Silicate Triangle graphs $SiT_{n}$ and presents an algorithm for computing their independent sets. In the later part, the MIPs are computed explicitly for a few iterations of the Silicate Triangle graphs. This section begins with the definition of the Silicate triangle graph. Note that our definition and notations are slightly different to those of Gayathiri and Manimaran (2024).

Definition 4 The Silicate Triangle graphs ( SiT in short) are defined inductively. The first iteration $SiT_{1}$ is an equilateral triangle with a vertex in the center (called the central vertex), and the central vertex is connected to all three vertices of the triangle. The vertices other than the center are called the outer vertices. Now assume, by induction, that $SiT_{n}$ has been constructed. Then, there are $3^{n}$ outer vertices in $SiT_{n}$ . To construct $SiT_{n+1}$ , we add a copy of $SiT_{1}$ for each outer vertex and identify the central vertex of $SiT_{1}$ with the outer vertex of $SiT_{n}$ . Subsequently, we call the outer vertices of each added copy of $SiT_{1}$ as the outer vertices of $SiT_{n+1}$ .

**FIGURE 2:**
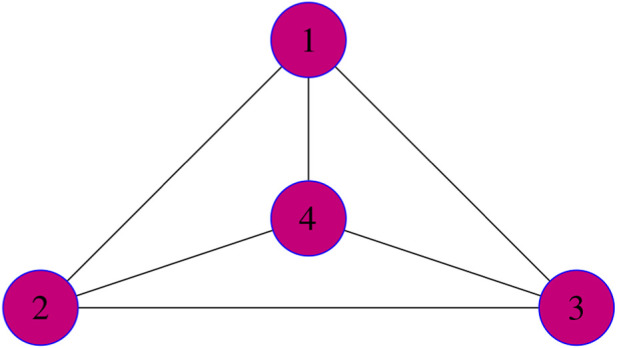
First iteration of Silicate Triangle $\left(SiT_{1}\right)$ .

**FIGURE 3:**
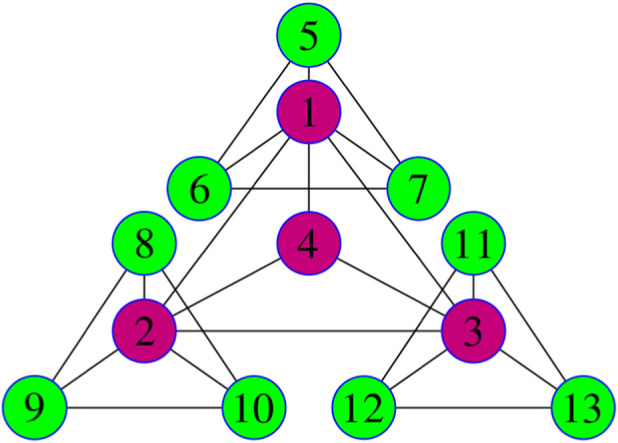
Second iteration of Silicate Triangle $\left(SiT_{2}\right)$ .

**FIGURE 4:**
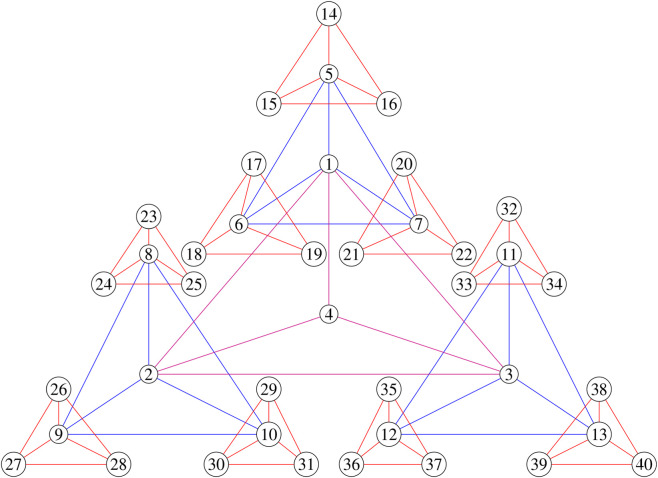
Third iteration of Silicate Triangle $\left(SiT_{3}\right)$ .

We draw the Silicate Triangle graphs for the first few iterations. Figure 2 describes the first iteration of the Silicate Triangle (*SiT*_1_), whereas Figures 3, 4 describe the second and third iterations of the Silicate Triangle (*SiT*_2_ and *SiT*_3_) respectively.

### 3.1 Vertices and edges

The following Lemma determines the number of vertices and edges of $SiT_{n}$ . For completeness, we supply the details of the proof using generating functions.

Lemma 1 We have $|V\left(SiT_{n}\right)|=\frac{3^{n+1}-1}{2}$ and $|E\left(SiT_{n}\right)|=3^{n+1}-3$ .

Proof. Let $v_{n}=|V\left(SiT_{n}\right)|$ , and let $F\left(z\right)=\sum_{n=1}^{\infty }v_{n}z^{n}$ be the generating function of $v_{n}$ . To construct $SiT_{n+1}$ from $SiT_{n}$ , we note that a copy of $SiT_{1}$ is associated with each outer vertex of $SiT_{n}$ so that the central vertex of the $SiT_{1}$ copy is identified with the outer vertex. Since there are $3^{n}$ outer vertices in $SiT_{n}$ , it follows that $v_{n}$ satisfies the recurrence relation

$$v_{n+1}=v_{n}+3\cdot 3^{n},\;\;v_{1}=4.$$ (1)

Multiplying both sides of Equation 1 by $z^{n+1}$ and summing, we obtain

$$\begin{array}{ll} \overset{\infty }{\underset{n=1}{\sum }}v_{n+1}z^{n} & =\overset{\infty }{\underset{n=1}{\sum }}v_{n}z^{n}+\overset{\infty }{\underset{n=1}{\sum }}3^{n+1}z^{n}\\ \Rightarrow \frac{1}{z}\overset{\infty }{\underset{n=1}{\sum }}v_{n+1}z^{n+1} & =\overset{\infty }{\underset{n=1}{\sum }}v_{n}z^{n}+\overset{\infty }{\underset{n=1}{\sum }}3^{n+1}z^{n}\\ \Rightarrow \frac{1}{z}\left(F\left(z\right)-v_{1}z\right) & =F\left(z\right)+3\left[\frac{1}{1-3z}-1\right]\\ \Rightarrow F\left(z\right) & =\frac{4z-3z^{2}}{\left(1-z\right)\left(1-3z\right)}. \end{array}$$

Using partial fraction decomposition, we obtain

$$F\left(z\right)=1-\frac{1}{2}\left(\frac{1}{1-z}\right)+\frac{3}{2}\left(\frac{1}{1-3z}\right).$$

Thus,

$$F\left(z\right)=2+\overset{\infty }{\underset{n=1}{\sum }}\left(\frac{3^{n+1}-1}{2}\right)z^{n}.$$

Hence, $v_{n}=\left(3^{n+1}-1\right)/2$ for n≥1 as required.

Let us take $e_{n}=|E\left(SiT_{n}\right)|$ , and let $G\left(z\right)=\sum_{n=1}^{\infty }e_{n}z^{n}$ be the generating function of $e_{n}$ , where $e_{n}$ satisfies the recurrence relation.

$$e_{n+1}=e_{n}+6\cdot 3^{n},\;\;e_{1}=6,$$

$$e_{n+1}=e_{n}+2\cdot 3^{n+1}.$$ (2)

Multiplying both sides of Equation 2 by $z^{n+1}$ and summing, we obtain

$$\begin{array}{ll} \overset{\infty }{\underset{n=1}{\sum }}e_{n+1}z^{n+1} & =\overset{\infty }{\underset{n=1}{\sum }}e_{n}z^{n+1}+6\overset{\infty }{\underset{n=1}{\sum }}3^{n}z^{n+1}\\ \Rightarrow \frac{1}{z}\overset{\infty }{\underset{n=1}{\sum }}e_{n+1}z^{n+1} & =\overset{\infty }{\underset{n=1}{\sum }}e_{n}z^{n}+6\overset{\infty }{\underset{n=1}{\sum }}3^{n}z^{n}\\ \Rightarrow \frac{1}{z}\left(G\left(z\right)-e_{1}z\right) & =G\left(z\right)+6\left[\frac{1}{1-3z}-1\right]\\ \Rightarrow G\left(z\right)-6z & =z\cdot G\left(z\right)+\frac{18\cdot z^{2}}{\left(1-3z\right)}\\ \Rightarrow G\left(z\right) & =\frac{6z}{\left(1-z\right)\left(1-3z\right)}. \end{array}$$

Using partial fraction decomposition, we obtain

$$G\left(z\right)=-3\left(\frac{1}{1-z}\right)+3\left(\frac{1}{1-3z}\right).$$

Thus,

$$G\left(z\right)=\overset{\infty }{\underset{n=1}{\sum }}\left(3^{n+1}-3\right)z^{n}.$$

Hence, $e_{n}=\left(3^{n+1}-3\right)$ for n≥1 as required.

### 3.2 Proposed algorithmic approach to compute independent sets

This subsection provides an algorithmic approach for obtaining the independent sets of $SiT_{n}$ from $SiT_{n-1}$ . Let $TV_{n-1}=\left\{v_{n-1,i}:1\leq i\leq 3^{n-1}\right\}$ denote the terminal vertices of $SiT_{n-1}$ . We also use $T_{n,i}$ , for $1\leq i\leq 3^{n-1}$ , to denote the copy of $SiT_{1}$ in $SiT_{n}$ associated with the vertex $v_{n-1,i}$ . Note that the terminal vertex $v_{n-1,i}$ in $SiT_{n-1}$ becomes a non-terminal vertex in $SiT_{n}$ , which serves as the central vertex of $T_{n,i}$ . For n≥2 , set $P_{n}≔I\left(SiT_{n-1}\right)\times \prod_{i=1}^{3^{n}}P\left(V\left(T_{n,i}\right)\right)$ . Define $S_{n}$ to be the subset of $P_{n}$ given by

$$S_{n}=\left\{\left(I,I_{i}:1\leq i\leq 3^{n-1}\right):|I_{i}|\leq 1\text{ and }|I_{i}|=1\text{ if and only if }v_{n-1,i}\notin I\right\}.$$

The next theorem proves that the set S describes all independent sets of $SiT_{n}$ .

Theorem 1 There exists a one to one correspondence between $I\left(SiT_{n}\right)$ and $S_{n}$ .

Proof. Let $I\in I\left(SiT_{n}\right)$ . Define $I_{0}≔I\cap V\left(SiT_{n-1}\right)$ and for $1\leq i\leq 3^{n-1}$ , define $I_{i}=I\cap V\left(T_{n,i}\right)$ . Define a map $f:I\left(SiT_{n}\right)\to S_{n}$ by

$$I\mapsto \left(I_{i}:0\leq i\leq 3^{n-1}\right).$$

It is easy to observe that the map f is well defined and injective. Since I is an independent set, to prove that it is surjective, let $\left(I_{i}:0\leq i\leq 3^{n-1}\right)\in S_{n}$ . If $I≔\cup_{i=0}^{3^{n-1}}I_{i}$ is not an independent set in $SiT_{n}$ , then there exists $1\leq i\leq 3^{n-1}$ such that $I_{0}$ and $I_{i}$ have adjacent vertices. However, this is a contradiction to the definition of $S_{n}$ . Therefore, the map is also surjective, and this completes the proof.

## 4 Construction of IP and MIP for iterations of $SiT_{n}$

In this section, we compute the IPs for first few iterations. We show some applications of Theorem 1 and compute the MIPs for iterations of the Silicate Triangle graph.

### 4.1 IP for $SiT_{1}$

The first iteration of Silicate Triangle graph (*SiT*_1_) is provided in Figure 2. $SiT_{1}$ is isomorphic to the complete graph $K_{4}$ , and there is only one independent subset with no elements and there are four independent sets with one element. Therefore, the IP of $SiT_{1}$ is given by

$$IP_{SiT_{1}}\left(x\right)=1+4x.$$

### 4.2 IP for $SiT_{2}$

We use Theorem 1 to determine the independent sets of $SiT_{2}$ . The second iteration of Silicate Triangle graph (*SiT*_2_) is provided in Figure 3. We carefully investigate the cases. Note that if I is an independent set of $SiT_{2}$ , then we must have $|I\cap TV_{1}|\leq 1$ .

1. There is exactly one independent set with cardinality 0, and there are 13 independent sets with cardinality 1.
2. Let I be an independent set of $SiT_{2}$ with cardinality 2. If $|I\cap TV_{1}|=0$ , then either I={a,b} for any $a\in T_{2,i}$ and $b\in T_{2,j}$ for i≠j or I contains the central vertex of the middle copy of $SiT_{1}$ and one other vertex from the outer triangles. In the former case, there are 3232=27 such choices and the number of choices from the latter case is 31×3=9 . If $|I\cap TV_{1}|=1$ , then without loss of generality, let $I\cap TV_{1}=\left\{v_{n-1,i}\right\}$ . Then, from Theorem 1, it is clear that $I_{i}=\emptyset$ . Therefore, in this case, we have 31×6=18 such choices for I . Thus, in this case, we have a total of 54 independent such sets.
3. Let I be an independent set of $SiT_{2}$ with cardinality 3. If $|I\cap TV_{1}|=0$ , then either I={a,b,c} where a,b,c come from different copies of $T_{2,i}$ or I contains the central vertex of the middle copy of $SiT_{1}$ and two other vertices from two distinct copies of the outer triangles. In the former case, there are $3^{3}=27$ such independent sets, and the number is 32×32=27 in the latter case. If $|I\cap TV_{1}|=1$ , then without loss of generality, let $I\cap TV_{1}=\left\{v_{n-1,i}\right\}$ . Then again, Theorem 1 implies that $I_{i}=\emptyset$ . In this case, $I=\left\{v_{n-1,i},b,c,\right\}$ , where b and c belong to different $T_{2,j}$ , neither of which is equal to $T_{2,i}$ . In particular, there are 3132=27 different independent sets. Thus, in this case, we have a total of 81 independent such sets.
4. Let I be an independent set of $SiT_{2}$ with cardinality 4. Note that by Theorem 1, the only way we can obtain such sets is that $|I\cap TV_{1}|=0$ . Therefore, I contains the central vertex of the middle copy of $SiT_{1}$ and three other vertices from each copy of the outer triangles. In particular, there are $3^{3}=27$ such sets.

According to Theorem 1, the highest number of elements in an independent subset of $SiT_{2}$ is 4. Therefore, the independence polynomial of $SiT_{2}$ is given by

$$IP_{SiT_{2}}\left(x\right)=1+13x+54x^{2}+81x^{3}+27x^{4}.$$

### 4.3 IP for $SiT_{3}$

We use Theorem 1 to determine certain independent sets of $SiT_{3}$ from the independent sets of $SiT_{2}$ counted in Section 4.2. We carefully investigate the cases. Note that if I is an independent set of $SiT_{3}$ , then $|I\cap TV_{2}|\leq 3$ , and vertices should come from different copies of $T_{3,i}$ .

1. There is exactly one independent set with cardinality 0, and there are 40 independent sets with cardinality 1.
2. Let I be an independent set of $SiT_{3}$ with cardinality 2. If I is an independent subset of $SiT_{2}$ , then there are 54 such sets. So, assume that I contains at least one vertex in the outer triangles. Therefore, we have $|I\cap TV_{2}|\leq 1$ .
  - First, assume that $|I\cap TV_{2}|=0$ . Then, either I is fully contained in the outer triangles or it contains a vertex from $SiT_{2}$ and a vertex from the outer triangles. In the former case, the total number of such independent sets is 92×32=324 and (13−9)×9×3=108 for the latter. So, the total number of such independent subsets is 432.
  - Assume that $|I\cap TV_{2}|=1$ . Then, according to Theorem 1, the other vertex of I must come from the outer triangles that are not adjacent to the vertex in $I\cap TV_{2}$ . The number of such triangles is 8. Therefore, total number of such independent sets is 91×8×3=216 .

Thus, the total number of independent sets of $SiT_{3}$ with cardinality 2 is 702. Similarly, we can compute the number of independent sets with higher cardinality. We write these numbers using SageMath for the reader’s convenience.

3. There are a total of 7,128 independent sets with cardinality 3.
4. There are a total of 46,440 independent sets with cardinality 4.

We can derive from Theorem 1 that the maximum cardinality an independent subset of $SiT_{3}$ can have is 10, which is also confirmed by SageMath (The SageD, 2024).

The computational time for the number of independent sets (here, the parameter is the iterative value n ) based on the parameters n is provided in Table 1. The table shows that the execution time increases with the iteration level, reflecting the increasing combinatorial complexity of the graph structure. The number of independent sets increases exponentially with the $n^{\text{th}}$ iteration of the construction, making the enumeration of independent sets more complicated. However, the independent sets associated with the $n^{\text{th}}$ iteration can be computed efficiently using well-established and computationally economical mathematical tool, thereby providing a practical and user-friendly approach for future large-scale analytical explorations.

**TABLE 1:** Execution time analysis for enumerating independent sets.

Iteration (n)	Execution time (in seconds)
1	30
2	60
3	120

### 4.4 Multivariate independence polynomial for $SiT_{1}$

For $SiT_{1}$ , the independent sets consist of the empty set and the sets containing a single vertex. Hence, the MIP is given by $MIP_{SiT_{1}}\left(\underline{x}\right)=1+x_{1}+x_{2}+x_{3}+x_{4}$ .

Using the binomial identity, we can compute its inverse as follows:

$$MIP_{SiT_{1}}{\left(\underline{x}\right)}^{-1}=\underset{m\geq 0}{\sum }{\left(-1\right)}^{m}{\left(x_{1}+x_{2}+x_{3}+x_{4}\right)}^{m}.$$

### 4.5 Multivariate independence polynomial for $SiT_{2}$

In this subsection, we shall compute the independent sets of $SiT_{2}$ explicitly. We shall use the arrangement of the vertices provided in Figure 4.

- The independent sets with exactly one element are given by:

$$\left\{\left\{1\right\},\left\{2\right\},\left\{3\right\},\left\{4\right\},\left\{5\right\},\left\{6\right\},\left\{7\right\},\left\{8\right\},\left\{9\right\},\left\{10\right\}\left\{11\right\},\left\{12\right\},\left\{13\right\}\right\}.$$

- Therefore, the degree one term of the multivariate independence polynomial is

$$\overset{13}{\underset{i=1}{\sum }}x_{i}.$$

- The independent sets with two elements are given by

$$\left\{10,1\right\},\left\{10,3\right\},\left\{10,4\right\},\left\{10,5\right\},\left\{10,6\right\},\left\{10,7\right\},\left\{10,11\right\},\left\{10,12\right\},\left\{10,13\right\},\left\{1,8\right\},\left\{1,9\right\},$$

$$\left\{1,11\right\},\left\{1,12\right\},\left\{1,13\right\},\left\{2,5\right\},\left\{2,6\right\},\left\{2,7\right\},\left\{2,11\right\},\left\{2,12\right\},\left\{2,13\right\},\left\{3,5\right\},\left\{3,6\right\},\left\{3,7\right\},$$

$$\left\{3,8\right\},\left\{3,9\right\},\left\{4,5\right\},\left\{4,6\right\},\left\{4,7\right\},\left\{4,8\right\},\left\{4,9\right\},\left\{4,11\right\},\left\{4,12\right\},\left\{4,13\right\},\left\{8,5\right\},\left\{9,5\right\},$$

$$\left\{11,5\right\},\left\{12,5\right\},\left\{13,5\right\},\left\{8,6\right\},\left\{9,6\right\},\left\{11,6\right\},\left\{12,6\right\},\left\{13,6\right\},\left\{8,7\right\},\left\{9,7\right\},$$

$$\left\{11,7\right\},\left\{12,7\right\},\left\{13,7\right\},\left\{8,11\right\},\left\{8,12\right\},\left\{8,13\right\},\left\{9,11\right\},\left\{9,12\right\},\left\{9,13\right\}.$$

- Therefore, the degree two term of the MIP is given by

$$\begin{array}{ll} & x_{1}x_{10}+x_{3}x_{10}+x_{4}x_{10}+x_{5}x_{10}+x_{6}x_{10}+x_{7}x_{10}+x_{10}x_{11}+x_{10}x_{12}+x_{10}x_{13}+x_{1}x_{8}+x_{1}x_{9}+\\ & x_{1}x_{11}+x_{1}x_{12}+x_{1}x_{13}+x_{2}x_{5}+x_{2}x_{6}+x_{2}x_{7}+x_{2}x_{11}+x_{2}x_{12}+x_{2}x_{13}+x_{3}x_{5}+x_{3}x_{6}+x_{3}x_{7}\\ & x_{3}x_{8}+x_{3}x_{9}+x_{4}x_{5}+x_{4}x_{6}+x_{4}x_{7}+x_{4}x_{8}+x_{4}x_{9}+x_{4}x_{11}+x_{4}x_{12}+x_{4}x_{13}+x_{5}x_{8}+x_{5}x_{9}+\\ & x_{5}x_{11}+x_{5}x_{12}+x_{5}x_{13}+x_{6}x_{8}+x_{6}x_{9}+x_{6}x_{11}+x_{6}x_{12}+x_{6}x_{13}\;x_{7}x_{8}+x_{7}x_{9}+x_{7}x_{11}+x_{7}x_{12}\\ & x_{7}x_{13}+x_{8}x_{11}+x_{8}x_{12}+x_{8}x_{13}+x_{9}x_{11}+x_{9}x_{12}+x_{9}x_{13}. \end{array}$$

- The independent sets with three elements are given by

$$\left\{1,10,11\right\},\left\{1,10,12\right\},\left\{1,10,13\right\},\left\{10,3,5\right\},\left\{10,3,6\right\},\left\{10,3,7\right\},\left\{10,4,5\right\},\left\{10,4,6\right\},$$

$$\left\{10,4,7\right\},\left\{10,11,4\right\},\left\{10,4,12\right\},\left\{10,4,13\right\},\left\{10,11,5\right\},\left\{10,12,5\right\},\left\{10,13,5\right\},\left\{10,11,6\right\},$$

$$\left\{10,12,6\right\},\left\{10,13,6\right\},\left\{10,11,7\right\},\left\{10,12,7\right\},\left\{10,13,7\right\},\left\{8,1,11\right\},\left\{8,1,12\right\},\left\{8,1,13\right\},$$

$$\left\{1,11,9\right\},\left\{1,12,9\right\},\left\{1,13,9\right\},\left\{2,11,5\right\},\left\{2,12,5\right\},\left\{2,13,5\right\},\left\{2,11,6\right\},\left\{2,12,6\right\},\left\{2,13,6\right\},$$

$$\left\{2,11,7\right\},\left\{2,12,7\right\},\left\{2,13,7\right\},\left\{8,3,5\right\},\left\{9,3,5\right\},\left\{8,3,6\right\},\left\{9,3,6\right\},\left\{8,3,7\right\},\left\{9,3,7\right\},$$

$$\left\{8,4,5\right\},\left\{9,4,5\right\},\left\{11,4,5\right\},\left\{12,4,5\right\},\left\{13,4,5\right\},\left\{8,4,6\right\},\left\{9,4,6\right\},\left\{11,4,6\right\},\left\{4,12,6\right\},$$

$$\left\{4,13,6\right\},\left\{8,4,7\right\},\left\{9,4,7\right\},\left\{11,4,7\right\},\left\{4,12,7\right\},\left\{4,13,7\right\},\left\{8,11,4\right\},\left\{8,4,12\right\},\left\{8,4,13\right\},$$

$$\left\{9,11,4\right\},\left\{9,4,12\right\},\left\{9,4,13\right\},\left\{8,11,5\right\},\left\{8,12,5\right\},\left\{8,13,5\right\},\left\{9,11,5\right\},\left\{9,12,5\right\},$$

$$\left\{9,13,5\right\},\left\{8,11,6\right\},\left\{8,12,6\right\},\left\{8,13,6\right\},\left\{9,11,6\right\},\left\{9,12,6\right\},\left\{9,13,6\right\},\left\{8,11,7\right\},$$

$$\left\{8,12,7\right\},\left\{8,13,7\right\},\left\{9,11,7\right\},\left\{9,12,7\right\},\left\{9,13,7\right\}.$$

- The degree three term of the MIP is given by

$$\begin{array}{ll} & x_{1}x_{10}x_{11}+x_{1}x_{10}x_{12}+x_{1}x_{10}x_{13}+x_{3}x_{5}x_{10}+x_{3}x_{6}x_{10}+x_{3}x_{7}x_{10}+x_{4}x_{5}x_{10}+x_{4}x_{6}x_{10}+x_{4}x_{7}x_{10}\\ & \;+x_{4}x_{10}x_{11}+x_{4}x_{10}x_{12}+x_{4}x_{10}x_{13}+x_{5}x_{10}x_{11}+x_{5}x_{10}x_{12}+x_{5}x_{10}x_{13}+x_{6}x_{10}x_{11}+x_{6}x_{10}x_{12}\\ & \;+x_{6}x_{10}x_{13}+x_{7}x_{10}x_{11}+x_{7}x_{10}x_{12}+x_{7}x_{10}x_{13}+x_{1}x_{8}x_{11}+x_{1}x_{8}x_{12}+x_{1}x_{8}x_{13}+x_{1}x_{9}x_{11}+x_{1}x_{9}x_{12}\\ & \;+x_{1}x_{9}x_{13}+x_{2}x_{5}x_{11}+x_{2}x_{5}x_{12}+x_{2}x_{5}x_{13}+x_{2}x_{6}x_{11}+x_{2}x_{6}x_{12}+x_{2}x_{6}x_{13}+x_{2}x_{7}x_{11}+x_{2}x_{7}x_{13}\\ & \;+x_{3}x_{5}x_{8}+x_{3}x_{5}x_{9}+x_{3}x_{6}x_{8}+x_{3}x_{6}x_{9}+x_{3}x_{7}x_{8}+x_{3}x_{7}x_{9}+x_{4}x_{5}x_{8}+x_{4}x_{5}x_{9}+x_{4}x_{5}x_{11}+x_{4}x_{5}x_{12}\\ & \;+x_{4}x_{5}x_{13}+x_{4}x_{6}x_{8}+x_{4}x_{6}x_{9}+x_{4}x_{6}x_{11}+x_{4}x_{6}x_{12}+x_{4}x_{6}x_{13}+x_{4}x_{7}x_{8}+x_{4}x_{7}x_{9}+x_{4}x_{7}x_{11}\\ & \;+x_{4}x_{7}x_{12}+x_{4}x_{7}x_{13}+x_{4}x_{8}x_{11}+x_{4}x_{8}x_{12}+x_{4}x_{8}x_{13}+x_{4}x_{9}x_{11}+x_{4}x_{9}x_{12}+x_{4}x_{9}x_{13}+x_{5}x_{8}x_{11}\\ & \;+x_{5}x_{8}x_{12}+x_{5}x_{8}x_{13}+x_{5}x_{9}x_{11}+x_{5}x_{9}x_{12}+x_{5}x_{9}x_{13}+x_{6}x_{8}x_{11}+x_{6}x_{8}x_{12}+x_{6}x_{8}x_{13}+x_{6}x_{9}x_{11}\\ & \;+x_{6}x_{9}x_{12}+x_{6}x_{9}x_{13}+x_{7}x_{8}x_{11}+x_{7}x_{8}x_{12}+x_{7}x_{8}x_{13}+x_{7}x_{9}x_{11}+x_{7}x_{9}x_{12}+x_{7}x_{9}x_{13}. \end{array}$$

- The independent sets with four elements are given by

$$\left\{10,11,4,5\right\},\left\{10,12,4,5\right\},\left\{10,13,4,5\right\},\left\{10,11,4,6\right\},\left\{10,4,12,6\right\},\left\{10,4,13,6\right\},$$

$$\left\{10,11,4,7\right\},\left\{10,4,12,7\right\},\left\{10,4,13,7\right\},\left\{8,11,4,5\right\},\left\{8,12,4,5\right\},\left\{8,13,4,5\right\},\left\{9,11,4,5\right\},$$

$$\left\{9,12,4,5\right\},\left\{9,13,4,5\right\},\left\{8,11,4,6\right\},\left\{8,4,12,6\right\},\left\{8,4,13,6\right\},\left\{9,11,4,6\right\},\left\{9,4,12,6\right\},$$

$$\left\{9,4,13,6\right\},\left\{8,11,4,7\right\},\left\{8,4,12,7\right\},\left\{8,4,13,7\right\},\left\{9,11,4,7\right\},\left\{9,4,12,7\right\},\left\{9,4,13,7\right\}.$$

- The degree four term of the MIP is given by

$$\begin{array}{ll} & x_{4}x_{5}x_{10}x_{11}+x_{4}x_{5}x_{10}x_{12}+x_{4}x_{5}x_{10}x_{13}+x_{4}x_{6}x_{10}x_{11}+x_{4}x_{6}x_{10}x_{12}+x_{4}x_{6}x_{10}x_{13}+\\ & x_{4}x_{7}x_{10}x_{11}+x_{4}x_{7}x_{10}x_{12}+x_{4}x_{7}x_{10}x_{13}+x_{4}x_{5}x_{8}x_{11}+x_{4}x_{5}x_{8}x_{12}+x_{4}x_{5}x_{8}x_{13}+\\ & x_{4}x_{5}x_{9}x_{11}+x_{4}x_{5}x_{9}x_{12}+x_{4}x_{5}x_{9}x_{13}+x_{4}x_{6}x_{8}x_{11}+x_{4}x_{6}x_{8}x_{12}+x_{4}x_{6}x_{8}x_{13}+\\ & x_{4}x_{6}x_{9}x_{11}+x_{4}x_{6}x_{9}x_{12}+x_{4}x_{6}x_{9}x_{13}+x_{4}x_{7}x_{8}x_{11}+x_{4}x_{7}x_{8}x_{12}+x_{4}x_{7}x_{8}x_{13}+\\ & x_{4}x_{7}x_{9}x_{11}+x_{4}x_{7}x_{9}x_{12}+x_{4}x_{7}x_{9}x_{13}. \end{array}$$

Therefore, the sum of all the homogeneous multivariate polynomials mentioned above is the multivariate independence polynomial $MIP_{SiT_{2}}\left(\underline{x}\right)$ , where $\underline{x}=\left(x_{i}:1\leq i\leq 13\right)$ .

The SiT graph of each higher iteration is constructed recursively from self-similar copies of the preceding iteration. As the iteration level increases, the number of self-similar copies increases, making the graph structure more complex; consequently, the computation of independent sets becomes more challenging. The computations of IPs and MIPs are recursive, asymptotic, and complex because the structural patterns are replicated through repeated iterations, giving rise to combinatorial structures in the resulting graph polynomials that retain symmetry while exhibiting increasing complexity. This combinatorial structure is inherited from the basic unit of the graph through recursive composition. With the increase in the iteration level, the number of vertices, edges, and admissible combinatorial configurations by the polynomial term increases systematically. For IPs, the coefficient terms exhibit structured growth patterns and scaling properties that arise directly from the asymptotic behavior, despite the increase in the algebraic complexity of higher-order iterations. For MIPs, distinct variables are assigned to the vertices according to their structural patterns and the recursive replication of vertex sets within the Silicate Triangle graph framework.

### 4.6 Analysis via graphical representation of IP

The graphical analysis of the fractal structure and its iterations $SiT_{n}$ for n=1,2 is presented in terms of the IP. In these graphical interpretations, the horizontal axis represents the variables of the polynomial, while the vertical axis shows the corresponding values of the computed IP for each iteration.

#### 4.6.1 Graphical representation for IP

**FIGURE 5:**
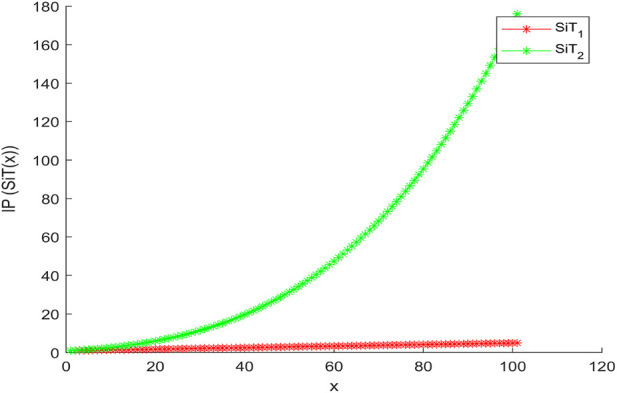
Univariate IP of $SiT_{n}$ (n = 1,2).

In this subsection, Figure 5 presents the graphical representation of the univariate IPs corresponding to $SiT_{1}$ and $SiT_{2}$ of the Silicate Triangle graph. The resulting curves exhibit smooth, continuous behavior that clearly reflects the structural progression of the IPs across successive iterations. Hence, this representation demonstrates the systematic growth and regularity of the univariate polynomial and the behavior of the IPs associated with the recursive silicate structures.

#### 4.6.2 Graphical representation for MIP

**FIGURE 6:**
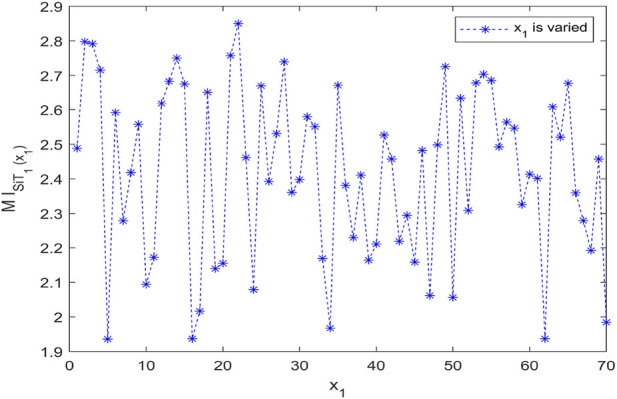
MIP of *SiT*_1_, when *x*_1_ is varied.

**FIGURE 7:**
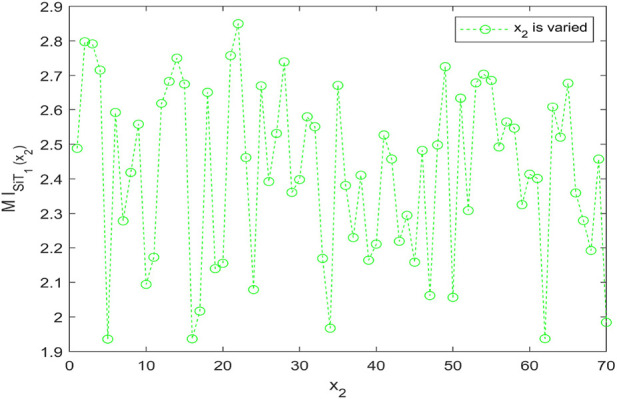
MIP of *SiT*_1_, when *x*_2_ is varied.

**FIGURE 8:**
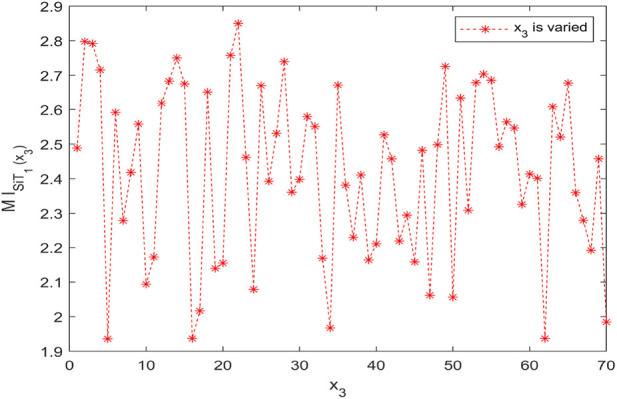
MIP of *SiT*_1_, when *x*_3_ is varied.

**FIGURE 9:**
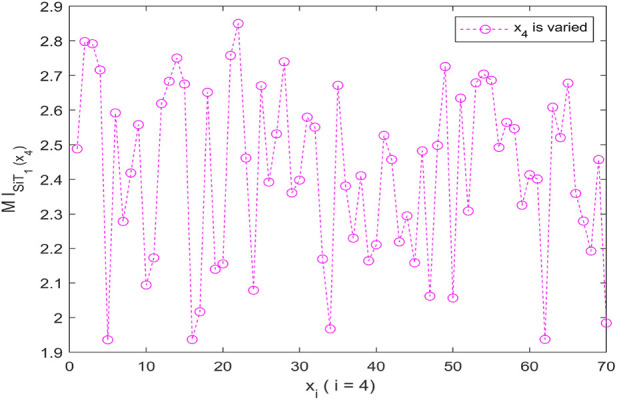
MIP of *SiT*_1_, when *x*_4_ is varied.

**FIGURE 10:**
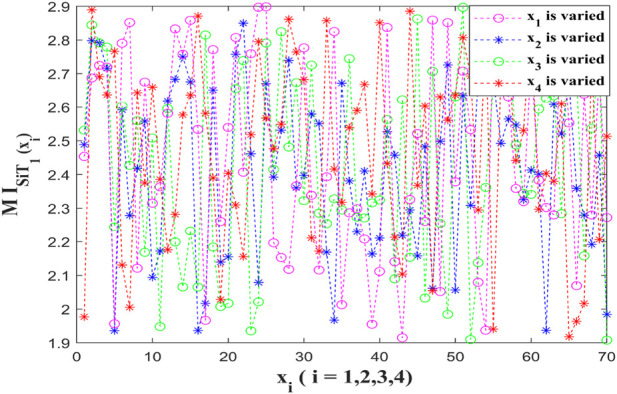
MIP of $SiT_{1}$ .

This subsection presents the graphical representation and analysis of MIP. Each graph is represented by varying $x_{i}$ while keeping *x*_*j*_’s constant for for all i and j such that i≠j . Using this type of representation, we illustrate the asymptotic behavior of this representative graph structure at each iteration. The polynomial exhibits systematic changes when $x_{1}$ is varied, while $x_{2}$ , $x_{3}$ , and $x_{4}$ are held constant, suggesting that the MIP is sensitive to the contributions of individual vertices. This behavior is consistently demonstrated in Figures 7, 8 with the variations in $x_{1}$ and $x_{2}$ having a significant effect on their MIP. Likewise, Figures 9, 10 shows how the structure of the polynomial changes as $x_{3}$ and $x_{4}$ vary, thereby highlighting the importance of interactions between the vertices of the multivariate case individually. The graphical representations corresponding to $SiT_{1}$ as sketched in Figure 6 clearly portrays that the MIP reflects the structural heterogeneity better than the univariate formulation. The polynomial changes significantly as the variables at the vertices change, reflecting the adjacency constraints and recursive combinatorial relationships within the graph. In comparison with all variables, $x_{1}$ , $x_{2}$ , and $x_{3}$ are the variables that display relatively higher variations and possess significant effects on the polynomial behavior.

**FIGURE 11:**
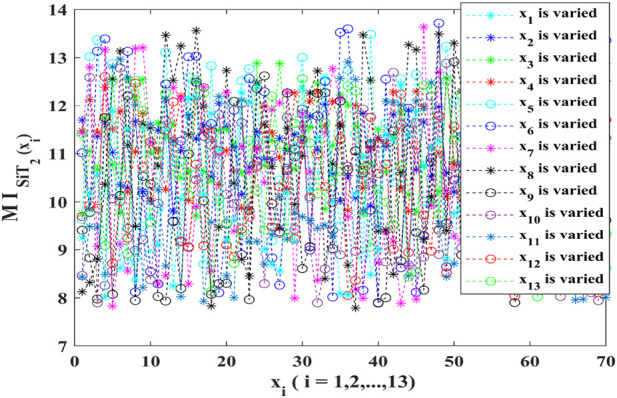
MIP of $SiT_{2}$

The graphical analysis of the MIP for the second iteration of the Silicate Triangle graph (*SiT*_2_), presented in Figure 11, demonstrates the influence of vertex-dependent interactions on the structural behavior of the polynomial. The variables $x_{1}$ , $x_{2}$ , $x_{3}$ , $x_{4}$ , $x_{5}$ , $x_{6}$ , $x_{7}$ , $x_{8}$ , $x_{9}$ , $x_{10}$ , $x_{11}$ , $x_{12}$ , and $x_{13}$ collectively contribute to the combinatorial formulation of the multivariate independence polynomial through recursive adjacency relations among the vertices. Among these variables, $x_{1}$ , $x_{2}$ , $x_{3}$ , $x_{5}$ , $x_{6}$ , $x_{7}$ , $x_{8}$ , $x_{9}$ , $x_{10}$ , $x_{11}$ , $x_{12}$ , and $x_{13}$ exhibit comparatively consistent variations, whereas the innermost vertex variable $x_{4}$ shows relatively lesser fluctuations, indicating it is more adjacent to other vertices. This behavior indicates that the outermost vertices contribute more significantly to the structural complexity and combinatorial growth of the polynomial than the innermost vertex. The fluctuations associated with the outermost vertices suggest greater adjacency interactions and increased participation in the formation of independent sets within the recursive Silicate Triangle network. Among all the variables, those associated with the outermost vertices exert the greatest influence on the MIP and effectively capture localized structural heterogeneity and recursive vertex dependencies through their graphical behavior. This indicates that the outermost vertices are structurally significant, possessing symmetry and revealing the existence of sparsity patterns among silicon-oxygen molecules in the fractal-like SiT graph framework.

A graph-theoretical approach of applying MIPs to a Silicate Triangle graph framework offers structural characteristics and molecular behavior in an illustrated manner. Unlike the classical independence polynomial, the multivariate formulation retains the structural adjacency information of the silicate network, allowing the distinction between boundary vertices, interior vertices, and recursively repeated vertex classes arising from its self-similar patterns. Since silicon atom exhibit adjacency behavior and oxygen atoms emphasize symmetry and exhibit consistent interactions, the MIP helps to encode the interplay between these atoms through chemical bonds and aids in understanding physiocochemical and structural properties such as connectivity, branching behavior, coordination patterns, and symmetry within the molecular framework. Since MIP preserves vertex-specific structural information and encodes recursive organization within complex molecular networks, these formulations reflect scaling behavior, hierarchical growth, and connectivity patterns of silicon and oxygen in fractal-like SiT graphs. Furthermore, the MIP acts as a refined mathematical descriptor that supports the analysis and prediction of structural complexity and physiochemical arrangement in self-similar silicon–oxygen-based molecular frameworks. Thus, MIPs can serve as predictive tools for modeling and analyzing intricate, self-similar chemical molecular structures, thereby enabling applications in material design and the optimization of molecular frameworks across a wide range of scientific and industrial domains.

The main limitation of this work lies in the computational complexity associated with higher-order iterations of the representative graphs. The number of independent sets increases exponentially and the number of multivariate polynomial terms grows rapidly in a combinatorial manner, making the analysis challenging due to runtime, memory, and representational issues and limiting the study to only a few iterations. However, further studies focusing on the development of higher-dimensional self-similar structures, as well as more complex generalized self-similar structures, could be carried out using scalable algorithms, thereby extending the applicability and theoretical development of multivariate graph polynomials in complex network modeling in the future.

## 5 Conclusion

By exploring graph polynomials derived from independent sets of fractal-like graphs, the representative Silicate Triangle graphs $SiT_{n}$ for ( n = 1,2 and 3) unveil a rich interplay between mathematical and chemical properties. By computing univariate IPs and MIPs, this article reveals and reflects the adjacency properties of molecular frameworks, particularly those involving silicon and oxygen atoms. Graphical analysis demonstrates the potential of MIPs in identifying symmetry, variable influence, and sparsity patterns, thereby contributing to the understanding of structural behavior in complex chemical networks. As the iterations of $SiT_{n}$ approach infinity, the asymptotic behavior of MIPs opens new avenues for research into the self-similarity of graphs and graph polynomial modeling. The computed results provide a valuable foundation for understanding the structural characteristics of this class of chemical graphs and their associated graph networks and also contributing valuable insights to the field of chemical graph theory and materials science. By bridging graph theory and molecular modeling, this work lays a robust foundational path for future investigations into the molecular design and analysis of structurally intricate self-similar materials in chemical science.

## Data Availability

The original contributions presented in the study are included in the article/supplementary material; further inquiries can be directed to the corresponding author.
